# Gastric Carcinoma with Exocrine and Neuroendocrine Components: A 16-Year Cohort Analysis and International Comparison

**DOI:** 10.3390/curroncol33090558

**Published:** 2026-09-15

**Authors:** Hu Ren, Zelin Wen, He Fei, Penghui Niu, Zefeng Li, Diliyaer Adili, Dongbing Zhao

**Affiliations:** National Cancer Center, National Clinical Research Center for Cancer, Cancer Hospital, Chinese Academy of Medical Sciences and Peking Union Medical College, Beijing 100021, China

**Keywords:** gastric cancer, neuroendocrine carcinoma, survival, MiNEN

## Abstract

**Background:** Gastric carcinoma with coexisting exocrine and neuroendocrine components (GC-EN) is rare and clinically aggressive, but its temporal pattern and prognostic impact remain poorly characterized. **Materials and Methods:** We retrospectively analyzed 1043 patients who underwent curative gastrectomy at our center between 2008 and 2024. Following tumor–node–metastasis staging and 1:2 pTNM stage matching, 882 patients were included; 294 had GC-EN, of which 175 had GNEC, and 588 had GC. Clinicopathological characteristics were compared, survival outcomes were assessed using Kaplan–Meier estimates and multivariate Cox regression, and the proportion of GNEC cases in our center and the U.S. surveillance, epidemiology, and end results (SEER) database were evaluated. Survival patterns of GNEC were further compared with published international cohorts using Engauge Digitizer software. **Results:** Among the patients with gastric cancer admitted for treatment, the proportion of NEC fluctuated between 4.01% and 4.38% (2008–2013) and finally dropped to 1.6% (2020–2021), whereas the SEER database showed a modest rise with a late dip (1.67% → 2.72% → 2.44%). The overall survival (OS) of patients with NEC appeared to improve at our center (1-/2-/3-year OS: 85.1%/59.6%/34% in 2008–2012 to 94.1%/87.5%/76.2% in 2021–2024), whereas that in the SEER database declined over the same periods. The 5-year OS of the two databases remained similar (42.7% vs. 41.0%). The 1, 2 and 5-year survival rates of NEC in the external cohorts were higher than those in the SEER and Berlin series; however, the 3-year survival rates were similar, with the Italian multicenter cohort performing the worst. The Kaplan–Meier analysis demonstrated significantly worse OS for GC-EN versus GC (*p* < 0.001); within GC-EN, the NEC subgroup (NEC ≥ 70%) had the poorest outcomes (*p* < 0.001), whereas mixed neuroendocrine-non-neuroendocrine neoplasm (MiNEN) and adenocarcinoma-dominant (AC-dominant) cases had similar outcomes as GC (*p* = 0.52, *p* = 0.81). In the multivariable Cox model, GC-EN histology remained independently associated with worse OS (HR = 1.49, 95% CI 1.15–1.92; *p* < 0.01). Metastatic lymph node burden and higher pTNM stage were associated with worse survival, whereas greater lymph node retrieval and age 40–65 years were protective. After adjustment, vascular invasion, tumor size, and location were not independently significant. **Conclusions:** Over the past 16 years at the National Cancer Center of China, the proportion of NEC cases decreased, and survival appeared to improve. NEC-dominant disease appeared to be the main driver of poor prognosis within GC-EN, whereas MiNEN and AC-dominant tumors showed survival patterns similar to GC in this cohort. These findings emphasize the need for GC-EN-specific risk stratification and tailored treatment strategies.

## Simple Summary

Some stomach cancers contain both ordinary gland-forming cancer cells and neuroendocrine cancer cells, but these mixed tumors are rare and are not well understood. Because previous studies were often small, doctors still lack clear evidence about how often these tumors occur, how their outcomes compare with usual stomach cancer, and whether the amount of neuroendocrine cancer affects prognosis. In this study, we reviewed patients treated at a large cancer center over 16 years and compared our findings with a United States population database. We found that patients with these mixed tumors had worse survival than patients with usual stomach cancer, especially when the neuroendocrine cancer component was dominant. These findings suggest that future studies and treatment guidelines should consider these tumors separately from usual stomach cancer and should pay attention to the proportion of neuroendocrine cancer when planning follow-up and treatment.

## 1. Introduction

Gastric cancer is one of the most common malignant tumors of the digestive system and remains a leading cause of cancer-related mortality worldwide. Despite advances in diagnosis and treatment, the prognosis of gastric cancer remains poor, particularly for histological subtypes with aggressive biological behavior [1,2,3]. Among these, gastric carcinoma with coexisting exocrine and neuroendocrine components (GC-EN) has attracted increasing attention. This subtype is characterized by the coexistence of adenocarcinoma and neuroendocrine carcinoma elements within the same tumor and is a highly malignant variant of gastric cancer [4,5,6].

Studies have suggested that GC-EN is more strongly associated with distinct clinicopathological features, including larger tumor size, deeper invasion, and more frequent lymph node metastasis than conventional gastric cancer (GC). Moreover, GC-EN often demonstrates more rapid progression and unfavorable survival outcomes [7,8,9,10,11,12]. However, owing to its rarity, most available evidence is derived from small, single-center cohorts or case reports, which limits the strength of current knowledge. The reported incidence and clinical significance of GC-EN vary substantially across studies, and survival patterns remain poorly defined.

The relationship between gastric neuroendocrine carcinoma (GNEC) and GC-EN has been inconsistently described. Although GNEC is commonly regarded as a major subtype of the GC-EN spectrum, its prognostic distinction from other GC-EN cases has not been clearly established [13,14,15]. Furthermore, there is a lack of comprehensive large-scale studies that systematically compare GC-EN with GC using robust statistical approaches. This limitation has hindered the development of evidence-based management strategies for this unique subtype.

To address these gaps, we retrospectively analyzed a large cohort of patients who underwent curative gastrectomies at our center. We evaluated the incidence, clinicopathological characteristics, and survival outcomes of GC-EN and compared them with those of GC. PTNM stage matching and long-term follow-up were used to minimize bias, and survival patterns were benchmarked against published international cohorts. Our findings provide a clearer understanding of GC-EN and evidence for its clinical management. This cohort study has been reported in line with the STROBE guidelines.

## 2. Methods

### 2.1. Study Design and Patient Selection

This retrospective study was conducted at the National Cancer Center (NCC) and included patients who underwent curative-intent gastrectomies for primary gastric malignancies between January 2008 and December 2024. In this study, GC-EN was used as a descriptive study term for gastric carcinoma with coexisting exocrine and neuroendocrine components, rather than as a newly proposed WHO diagnostic entity. NEC was defined according to the WHO classification as a poorly differentiated, high-grade neuroendocrine carcinoma with neuroendocrine morphology and immunohistochemical evidence of neuroendocrine differentiation. At our institution, the diagnostic cut-off for high-grade NEC was a Ki-67 index >20% and/or a mitotic count >20 per 2 mm^2^, interpreted together with tumor morphology. According to the 2019 WHO classification, mixed neuroendocrine-non-neuroendocrine neoplasm (MiNEN) requires both neuroendocrine and non-neuroendocrine components to account for at least 30% of the tumor. To evaluate whether the proportion of the neuroendocrine carcinoma component was associated with prognosis, GC-EN cases were further stratified into three groups according to the estimated NEC component: NEC-dominant tumors (NEC component ≥ 70%), WHO-defined MiNENs (30% < NEC component < 70%; previously referred to as MANEC), and adenocarcinoma-dominant tumors with neuroendocrine differentiation (NEC component ≤ 30%). NEC was defined according to the WHO classification as a poorly differentiated, high-grade neuroendocrine carcinoma with neuroendocrine morphology and immunohistochemical evidence of neuroendocrine differentiation. Neuroendocrine differentiation was confirmed using immunohistochemical markers, including synaptophysin, chromogranin A, CD56, and/or INSM1, according to the institutional pathology records. Patients with distant metastases at diagnosis, incomplete clinicopathological or follow-up data, or those lost to follow-up were excluded. Follow-ups were performed via scheduled telephone interviews and medical record review. The observation period was capped at 5 years postoperatively, and patients who were alive at 5 years were censored at that time. Patients or the public were not involved in the design, conduct, reporting, or dissemination plans of our research.

### 2.2. Patient Selection in the Surveillance, Epidemiology, and End Results Database

The Surveillance, Epidemiology, and End Results (SEER) database (www.seer.cancer.gov (accessed on 5 August 2025)) is a nationally representative cancer registry in the U.S. that covers approximately 35–48% of the U.S. population. For this study, we utilized the “SEER Research Plus Data, 18 Registries, Nov 2024 Sub (2000–2022)” to extract clinicopathological information of patients with GNEC and GC. Patients with GC were identified using the primary site code “C16.0–C16.9” (stomach) and the corresponding histology codes based on the International Classification of Diseases for Oncology, Third Edition (ICD-O-3): 8142 (linitis plastica), 8143 (superficial spreading adenocarcinoma), 8144 (adenocarcinoma, intestinal type), 8145 (carcinoma, diffuse type), 8210 (adenocarcinoma in adenomatous polyp), 8211 (tubular adenocarcinoma), 8214 (parietal cell carcinoma), 8221 (adenocarcinoma in multiple adenomatous polyps), 8255 (adenocarcinoma with mixed subtypes), 8260 (papillary adenocarcinoma, NOS), 8261 (adenocarcinoma in villous adenoma), 8262 (villous adenocarcinoma), 8263 (adenocarcinoma in tubulovillous adenoma), 8290 (oxyphilic adenocarcinoma), 8310 (clear cell adenocarcinoma, NOS), 8323 (mixed cell adenocarcinoma), 8410 (sebaceous adenocarcinoma), 8441 (serous cystadenocarcinoma, NOS), 8480 (mucinous adenocarcinoma), 8481 (mucin-producing adenocarcinoma), 8490 (signet ring cell carcinoma), 8503 (intraductal papillary adenocarcinoma with invasion), 8560 (adenosquamous carcinoma), 8570 (adenocarcinoma with squamous metaplasia), 8574 (adenocarcinoma with neuroendocrine differentiation), and 8576 (hepatoid adenocarcinoma). We also identified patients with GNEC with the primary site code as “C16.0–C16.9, stomach” and the following ICD-O-3 histology codes: 8013 (large cell neuroendocrine carcinoma), 8041 (small cell carcinoma, NOS), 8044 (small cell carcinoma), 8246 (neuroendocrine carcinoma, NOS). Patients with other malignancies and those without follow-up information were excluded. To improve comparability with the institutional cohort, we restricted the SEER cohort to patients without distant metastasis at diagnosis. Non-metastatic disease was identified using the SEER-derived M-stage variable applicable to each diagnosis year, including Derived EOD 2018 M Recode (2018+) = M0 for cases diagnosed from 2018 onward. We also included only patients who underwent surgery for the primary gastric tumor, defined by RX Summ–Surg Prim Site (1998+) codes 30, 31, 32, 33, 40, 41, 42, 50, 51, 52, 60, 61, 62, 63, and 80. Finally, 1783 patients with GNEC and 76,263 patients with GC were identified from 2008–2022.

### 2.3. Clinical and Pathological Data

The demographic and clinical information collected included sex, age at diagnosis, tumor location, tumor size, and postoperative complications. Pathological features recorded comprised histological type, tumor–node–metastasis (TNM) stage according to the 8th edition of the American Joint Committee on Cancer (AJCC) staging system, lymph node involvement, perineural invasion, and vascular invasion. The tumor location was categorized as proximal (cardia, fundus, or body), distal (antrum or pylorus), or involving the entire stomach. Overall survival (OS) was defined as the interval from surgery to death from any cause, expressed in months, with follow-up censored at 5 years postoperatively.

### 2.4. Proportion Trend Analysis

The temporal trend analysis was performed separately from the survival cohort. For each two-year calendar period from 2008 to 2021, the numerator was the number of gastric neuroendocrine carcinoma (NEC) cases, and the denominator was the number of all primary gastric cancer (GC) cases recorded in the same data source and period. Therefore, the denominator was not the 1043-patient survival cohort and was not limited to patients included in the survival analysis. The NCC trend dataset included 151 NEC cases and 7585 GC cases from 2008 to 2021. The corresponding SEER trend dataset included 1668 NEC cases and 71,253 GC cases from 2008 to 2021. Although the SEER extraction described in Section 2.2 included 1783 NEC cases and 76,263 GC cases through 2022, the trend analysis excluded 2022 to maintain complete two-year intervals and to use the same calendar window as the NCC trend analysis.

### 2.5. International Survival Comparison

Published survival curves from selected international cohorts were digitized using Engauge Digitizer to provide descriptive external context. Because individual patient data and numbers at risk were not consistently available, no formal statistical comparison or confidence interval reconstruction was performed. Therefore, these digitized data were interpreted only as a descriptive, non-statistical summary.

### 2.6. Statistical Analysis

PTNM stage matching at a 1:2 ratio was applied to minimize baseline differences between the GC-EN and the comparison groups (GC). Categorical variables are summarized as counts and percentages and compared using the chi-square or Fisher’s exact test, as appropriate. Continuous variables were expressed as means ± standard deviation or medians with interquartile ranges and compared using Student’s t-test or the Mann–Whitney U test. Overall survival curves were generated using the Kaplan–Meier method and differences between groups were evaluated using the log-rank test. Median follow-up was estimated using the reverse Kaplan–Meier method, with death treated as censoring. Numbers at risk were displayed below the Kaplan–Meier curves. The proportional hazards assumption for Cox regression models was assessed using Schoenfeld residuals. Pairwise survival comparisons among histologic subgroups were performed using log-rank tests, with Bonferroni correction applied to adjust for multiple comparisons. Univariate and multivariate Cox proportional hazards regression models were used to identify independent prognostic factors, and hazard ratios (HRs) and 95% confidence intervals (CIs) were reported. The number of retrieved lymph nodes and metastatic lymph nodes were entered into Cox models as continuous variables; their HRs represent the change in mortality hazard per one additional lymph node. Statistical significance was defined as a two-sided *p*-value of <0.05. All analyses were performed using R software (version 4.3.1).

## 3. Results

### 3.1. Baseline Characteristics

**Figure 1:**
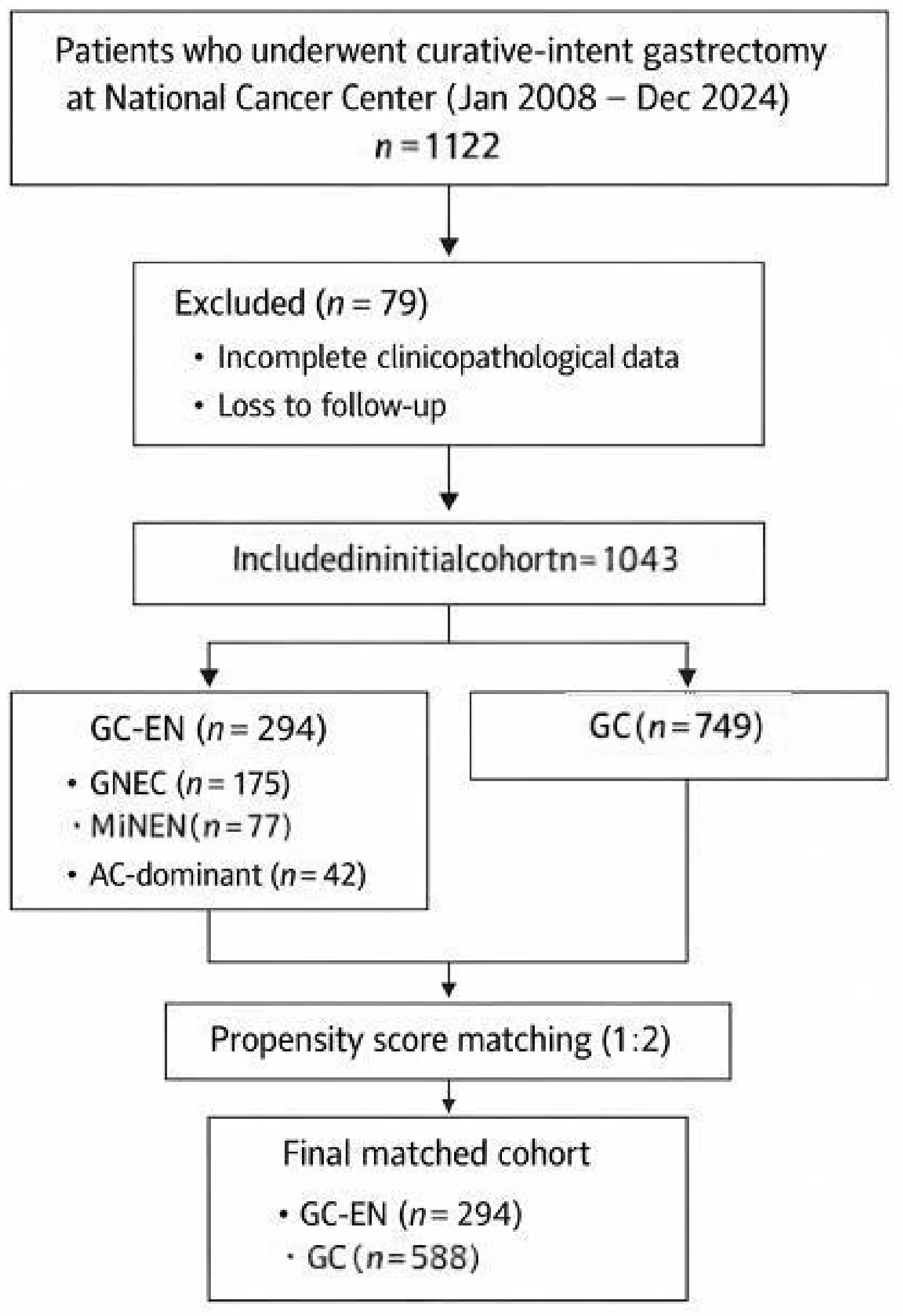
Flow diagram.

A total of 1122 patients were initially identified, and after excluding those with incomplete clinicopathological data or loss to follow-up, 1043 patients were ultimately included in the final analysis. After applying the pTNM stage matching in a 1:2 ratio, 882 patients were eligible for the analysis in Figure 1: 294 with GC-EN, 175 with GNEC, 77 with MiNEN, 42 who were AC-dominant, and 588 with GC (Before matching, it was 749). The baseline demographic and clinicopathological characteristics are summarized in Table 1.

Sex and age differed between the groups. The GC-EN group included more men (85% vs. 75%, *p* < 0.01) and more patients aged >65 years (104 vs. 53; *p* < 0.01). Tumor sizes > 4 cm were more frequently observed in the GC-EN group (*p* = 0.03). Regarding tumor locations, the GC-EN group had more proximal tumors (63% vs. 33%; *p* < 0.01). Nodal disease burden was similar in both groups. The mean number of positive nodes was 5.90 ± 9.37 vs. 5.03 ± 7.97 (*p* = 0.8). The GC-EN group had more nodes examined (mean 33.02 ± 15.74 vs. 29.52 ± 15.09; *p* < 0.01). Regarding the histology, signet ring cell components were more frequent in the GC group than in the GC-EN group (26% vs. 6.5%; *p* < 0.01), and HER2 amplification also differed significantly between the groups (*p* < 0.01). The pathological stage distributions were similar. The P stage did not differ between the groups (*p* > 0.9). The GC-EN group showed a higher proportion of vascular invasion (56% vs. 38%; *p* < 0.01). Perineural invasion was similar between groups (*p* = 0.6). The incidence of postoperative complications was comparable between the two groups (10% vs. 9.2%; *p* = 0.6).

### 3.2. Yearly Proportion of Nec Cases Among Gc Cases (NCC vs. SEER)

**Figure 2:**
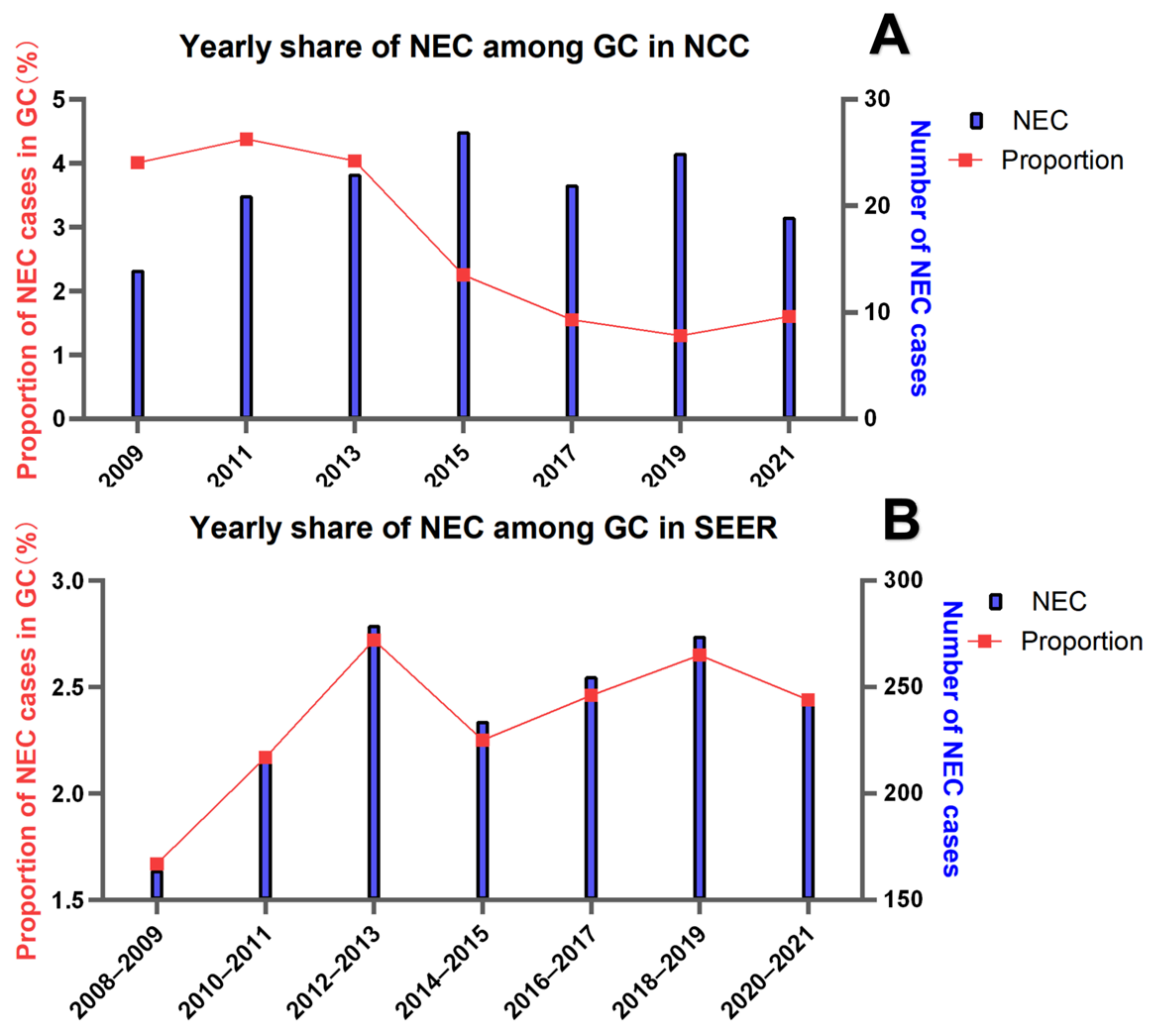
Institutional incidence of NEC cases in NCC and SEER databases. (**A**) The proportion of NEC cases in GC at the NCC center; (**B**) the proportion of NEC cases in the SEER database. NEC, neuroendocrine carcinoma; NCC, National Cancer Center; SEER, surveillance, epidemiology, and end results; GC, gastric cancer.

Our center and the SEER database showed different curves for the proportion of NEC cases among GC cases (Figure 2 and Supplemental Table S1). Our center showed a clear downward trend in Figure 2A. The share of NEC in GC fluctuated from 4.01% in 2008–2009 to 4.04% in 2012–2013, decreased to 1.3% in 2018–2019, then increased slightly to 1.6% in 2020–2021. The SEER data showed a slow rise, followed by a small dip. The proportion increased from 1.67% in 2008–2009 to 2.72% in 2012–2013 and decreased to 2.44% in 2020–2021. In short, our center showed a steady decline, whereas SEER showed a small increase with minor fluctuations in Figure 2B. Our center showed a higher proportion of NEC cases in the early periods and fell below that in the SEER data in the later period.

### 3.3. Survival Analysis of Nec by Time Period and Overall Trend (NCC vs. SEER)

**Figure 3:**
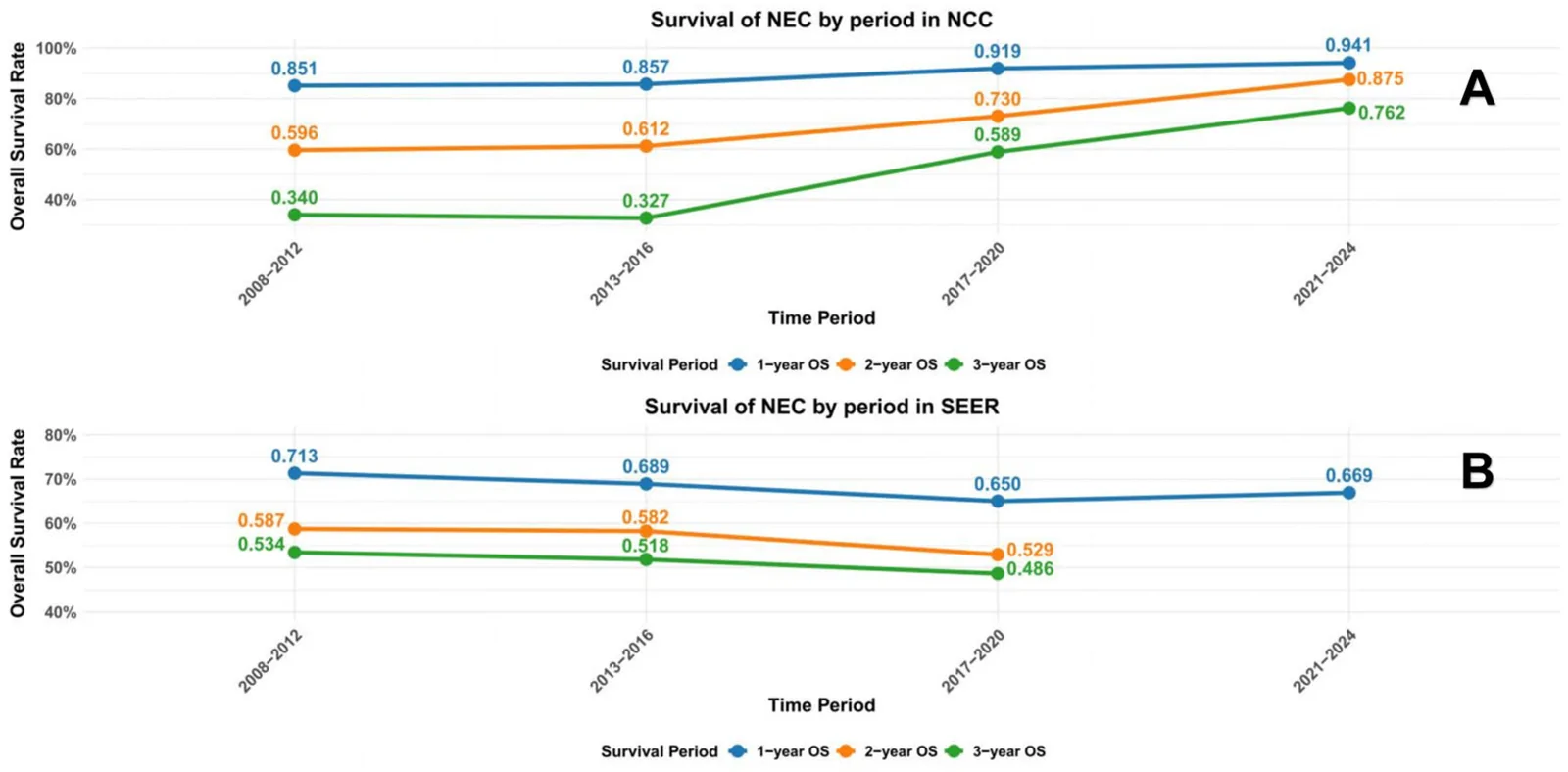
Survival analysis of NEC by time period (NCC vs. SEER). (**A**) Survival analysis of NEC cases by time period in the NCC database; (**B**) survival analysis of NEC cases by time period in the SEER database. NEC, neuroendocrine carcinoma; NCC, National Cancer Center; SEER, surveillance, epidemiology, and end results.

Survival curves for NEC differed (Figure 3 and Supplemental Table S2): our center showed steady gains in OS in the Figure 3A. From 2008 to 2012 and 2021 to 2024, the 1-year OS rose from 85.1% to 94.1%; 2-year OS, 59.6% to 87.5%; and 3-year OS, 34% to 76.2%. This increase was consistent across all the periods. The SEER data showed small declines in the Figure 3B, followed by a slight uptick. The 1-year OS moved from 71.3% in 2008–2012 to 66.9% in 2021–2024. The 2-year OS fell from 58.7% to 52.9% by 2017–2020. The 3-year OS fell from 53.4% to 48.6% by 2017–2020. The later 2-year and 3-year values were not available, and the gap between the groups widened over time. The 1-year OS gap grew from 13.8 points in 2008–2012 to 27.2 points in 2021–2024. The 2-year OS gap grew from 0.9 points to 20.1 points by 2017–2020. In 2008–2012, the 3-year OS was approximately 19.4 percentage points higher in the SEER database than at our center. By 2017–2020, this pattern had reversed, with the 3-year OS favoring our center by 10.3 percentage points.

Our center showed a higher OS for NEC than the SEER database (Table 2). This gap decreased over longer follow-up periods. The 3-year OS rate was slightly lower than that reported in the SEER database. Both cohorts had similar 5-year OS rates. The 1-, 2-, 3-, and 5-year OS rates were 88.6%, 68.3%, 44.9%, and 42.7%, whereas the OS rates of the SEER data were 65.5%, 53.2%, 47.8%, and 41.0%, respectively.

### 3.4. Nec Survival—International Comparisons

**Figure 4:**
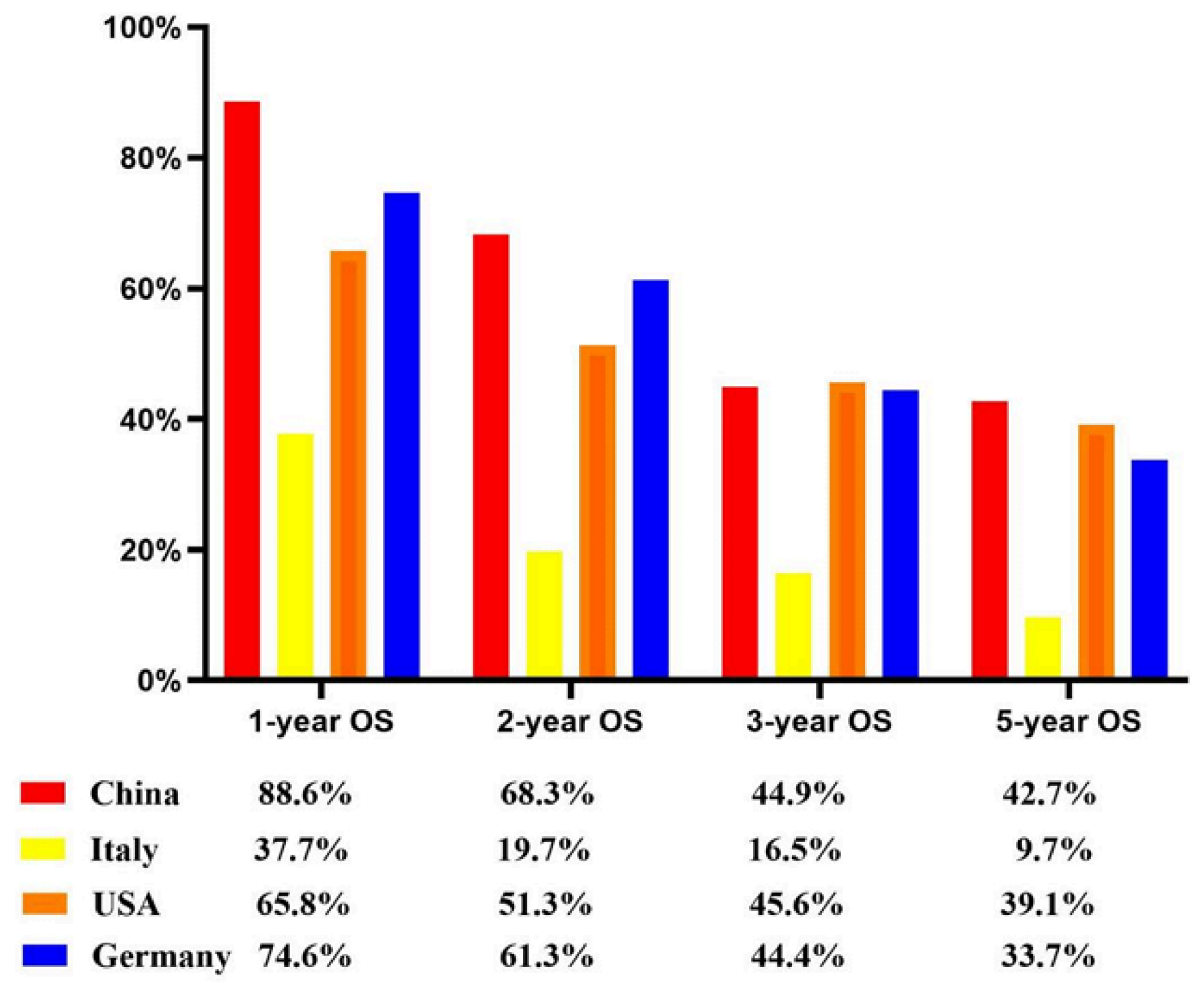
The survival of patients with NEC compared to those with NCC and the international community.

Our center showed higher early survival in NEC than the international cohorts (Figure 4 and Supplemental Table S3). Compared with that in another study from the SEER data [16], the survival rate in our center was higher at 1–2 years (88.6% vs. 65.8%, 68.3% vs. 51.3%) but slightly fell below the 3-year survival rate (44.9% vs. 45.6%) and was higher at 5 years (42.7% vs. 39.1%). Compared with the Berlin cohort [17], our center had higher rates at 1, 2, 3, and 5 years (88.6% vs. 74.6%, 68.3% vs. 61.3%, 44.9% vs. 44.4%, 42.7% vs. 33.7%). The Italian multicenter (Rome–Parma) cohort showed the poorest survival [18], with 1, 2, 3, and 5 year OS of 37.7%, 19.7%, 16.5%, and 9.7%, respectively. These data indicate a clear split: our center performed better in the short term.

### 3.5. Survival Outcomes

**Figure 5:**
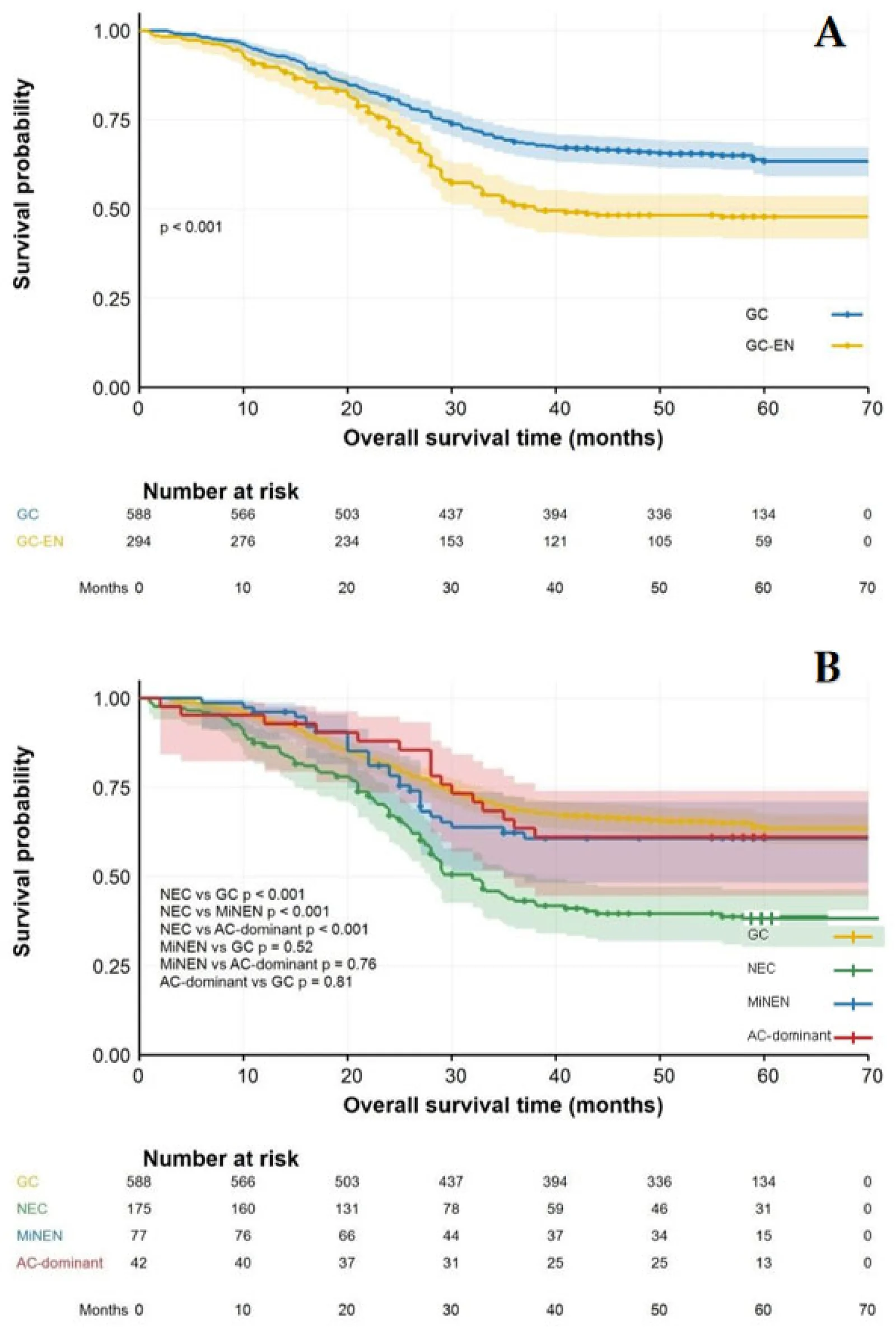
Comparison of survival between the GC-EN and GC groups in the NCC database. (**A**) Comparison of survival between the GC-EN and GC groups; (**B**) comparison of survival between the NEC, MiNEN, AC-dominant, and GC groups. Note: NEC (NEC component ≥70%), MiNEN (NEC and AC component > 30%), AC-dominant (NEC < 30%). NEC, neuroendocrine carcinoma;; GC, gastric cancer; MiNEN, 30% < NEC component < 70%; previously referred to as MANEC; AC-dominant, adenocarcinoma-dominant; GC-EN, gastric carcinoma with coexisting exocrine and neuroendocrine components.

The Kaplan–Meier survival analysis showed a lower OS for GC-EN than that for GC (Figure 5A). The separation appeared early and remained clear, and the differences were significant (*p* < 0.001,log-rank test). We divided GC-EN by the NEC fraction (Figure 5B). The NEC group showed the worst survival among the four groups (*p* < 0.01,log-rank test). The MiNEN, AC-dominant, and GC groups were similar (*p* = 0.52, *p* = 0.76, *p* = 0.81,log-rank test). Median follow-up in the matched cohort was 58.0 months by the reverse Kaplan–Meier method. In Figure 5A, the GC and GC-EN groups had 208/588 and 143/294 deaths, respectively; median OS was not reached in the GC group and was 38.0 months in the GC-EN group. Because GC-EN represents a heterogeneous category, we further evaluated the survival outcomes of NEC, MiNEN, and AC-dominant tumors separately in Figure 5B. In Figure 5B, deaths occurred in 208/588 GC, 99/175 NEC, 28/77 MiNEN, and 16/42 AC-dominant patients; median OS was not reached for GC, MiNEN, and AC-dominant tumors and was 32.0 months for NEC. The median follow-up was 58.0 months for GC, 59.0 months for GC-EN, 59.0 months for NEC, 59.0 months for MiNEN, and 60.0 months for AC-dominant tumors.

### 3.6. Cox Regression Analysis

Univariate and multivariate Cox proportional hazards analyses revealed several findings (Table 3). In univariable Cox proportional hazards models, compared with GC, GC-EN conferred a higher hazard ratio for shorter OS (HR = 1.66, 95% CI 1.34–2.05; *p* < 0.01). Additionally, age 40–65 years was associated with better OS compared with age <40 years (HR = 0.35, 95% CI 0.19–0.62; *p* < 0.01), whereas tumor size ≥ 4.0 cm was associated with shorter OS (HR = 1.46, 95% CI 1.18–1.82; *p* < 0.01). Distal tumor location showed a lower hazard ratio compared with proximal tumor location (HR = 0.78, 95% CI 0.62–0.97; *p* = 0.03). Additional lymph nodes retrieved were associated with a lower hazard ratio (HR = 0.99, 95% CI 0.98–1.00; *p* < 0.01), whereas additional metastatic lymph nodes jeopardized postoperative survival (HR = 1.03, 95% CI 1.02–1.04; *p* < 0.01). Higher pathologic stage was associated with worse outcomes (stage II vs. I: HR = 4.20, 95% CI 2.02–8.47; *p* < 0.01; stage III vs. I: HR = 8.07, 95% CI 3.99–16.30; *p* < 0.01), and vascular invasion was linked to higher mortality (HR = 1.28, 95% CI 1.04–1.58; *p* = 0.02). Sex, age > 65 years, signet-ring status, HER2 score, and perineural invasion showed no association with OS.

In the multivariate model, GC-EN remained an independent poor prognostic factor for OS (HR = 1.49, 95% CI 1.15–1.92; *p* < 0.01). Tumor size was not statistically significant after adjustment (HR = 1.06, 95% CI 0.85–1.34; *p* = 0.59). The number of metastatic lymph nodes diminished survival outcomes (HR = 1.03, 95% CI 1.02–1.05; *p* < 0.01), whereas a greater number of retrieved lymph nodes was protective (HR = 0.98, 95% CI 0.97–0.99; *p* < 0.01). Age 40–65 years remained protective compared with age < 40 years (HR = 0.40, 95% CI 0.22–0.72; *p* < 0.01). Higher pathologic stage had a significant direct impact on patient survival (stage II vs. I: HR = 4.54, 95% CI 2.17–9.50; *p* < 0.01; stage III vs. I: HR = 7.35, 95% CI 3.58–15.10; *p* < 0.01). After adjustment, vascular invasion, tumor size, and tumor location were not statistically significant. The proportional hazards assumption was not violated for the multivariable Cox model according to Schoenfeld residual testing (global chi-square = 5.420, df = 9, *p* = 0.796).

## 4. Discussion

In this large cohort, patients with GC-EN showed significantly worse survival than those with GC. GC-EN remained an independent adverse prognostic factor after adjustment. Metastatic lymph node burden was a strong predictor of poor outcomes, whereas a higher number of retrieved nodes and age 40–65 years were protective. The pathological stage was significantly associated with poor prognostic outcomes. Tumor size was a significant factor in the univariate analysis but lost significance after adjustment, suggesting that its prognostic effect is largely mediated by nodal status, corroborating previous population-based studies [19].

The apparent decline in the institutional proportion of NEC should be interpreted cautiously as a denominator effect rather than a definite decline in disease occurrence. As shown in Supplementary Table S1, the absolute number of NEC cases remained relatively stable across two-year periods, whereas the number of surgically treated adenocarcinoma cases increased substantially. Therefore, the declining proportion of NEC at our center likely reflects expansion of the surgical referral denominator and changes in case mix, rather than a true reduction in NEC incidence. Notable variations exist in NEC survival rates across ethnic groups [20]. Our findings confirmed that GC-EN confers inferior survival compared with GC, which is consistent with prior studies in Western cohorts. GC-EN is characterized by rapid proliferation, high mitotic activity, and early systemic spread, and genomic alterations in TP53 and RB1 have also been reported [21,22]. Although CD56 expression was not analyzed in the present cohort, previous studies have reported that CD56 negativity is associated with poorer prognosis in GC-EN. This association may be related to tumor–immune interactions involving NK-cell biology; however, the underlying mechanism remains unclear [23,24,25]. These biological features explain the aggressive clinical behavior and suggest that surgery alone is insufficient. The integration of systemic therapies tailored to neuroendocrine biology may be essential to improve outcomes.

In China, NEC survival appeared to improve compared with the SEER database. However, this trend should be interpreted as a descriptive observation rather than evidence that screening, healthcare coverage, or treatment advances directly caused the survival improvement, because patient-level data on screening history, diagnostic pathway, treatment strategy, and perioperative care were not collected. Potential explanations include differences in referral patterns, diagnostic timing, surgical quality, perioperative management, case mix, and treatment heterogeneity [26,27]; however, these factors cannot be directly verified in the present dataset. In addition, the 5-year survival rate in China was similar to that in the U.S., and the gap between China and Germany narrowed. Overall, NEC remains associated with poor survival outcomes worldwide, which may be related to insufficient understanding of its metastatic and recurrent mechanisms, immune microenvironment, and the lack of effective treatment strategies [28,29,30,31,32].

Patients with GC-EN aged 40–65 years had better survival than those aged <40 years in this cohort. Similar age-related trends have been described in gastric cancer, where very young patients may present with distinct clinicopathological and molecular features [31,33]. However, the subgroup aged <40 years included only a limited number of patients, and molecular or hereditary testing data were not available in this study. Therefore, this age-related association should be interpreted cautiously and requires validation in larger datasets with molecular annotation.

Both the number of metastatic and retrieved nodes were powerful prognostic determinants of GC-EN. Each additional metastatic node increased mortality risk, whereas more retrieved nodes improved survival. This dual effect reflects tumor aggressiveness and surgical quality. Adequate lymphadenectomy not only ensures precise staging but may also remove microscopic disease. Our results reinforce the importance of high-quality nodal dissection in GC-EN [34,35,36].

Pathological stage and vascular invasion were associated with worse GC-EN outcomes. This suggests that the prognostic value is mediated by the risk of metastasis and recurrence. For example, in higher stages, especially in cases of lymph node metastasis or vascular invasion, the risk of recurrence increases significantly [34]. The presence of proximal tumors and a larger tumor size may be associated with an unfavorable prognosis; however, the regression analysis did not reveal any statistically significant differences. Furthermore, different GC-ENs may exhibit varying degrees of malignancy at different locations [37,38].

Traditional histopathological factors, such as perineural invasion, HER2 status, and signet ring morphology, were not associated with survival in GC-EN. Although these markers are prognostic markers for conventional GC, their influence may be overshadowed by the dominant neuroendocrine component. This indicates that GC-EN requires dedicated prognostic models rather than direct extrapolation from adenocarcinoma [39,40,41].

This study had several limitations. Although this is one of the largest single-center series of GC-EN, subgroup analyses are limited by the rarity of the cases. Treatment heterogeneity, particularly in chemotherapy regimens, cannot be completely controlled. In addition, the lack of molecular data restricts mechanistic interpretation. External validation in multicenter cohorts would further strengthen the generalizability of these results. Finally, survival estimates for the most recent NCC period should be interpreted cautiously because the NCC cohort was followed through 2025, whereas SEER data were available only through 2022, and the 3-year OS estimate for 2021–2024 was immature due to limited follow-up maturity. Because of the retrospective design, detailed patient-level Ki-67 values and small-cell versus large-cell NEC subtype information were not systematically available for all cases and therefore were not included in the statistical analysis. Because of the limited number of patients and events in the MiNEN and AC-dominant subgroups, multivariable Cox regression stratified by each GC-EN subtype was not performed; instead, subgroup survival was presented descriptively in Figure 5B.

Future studies should integrate molecular profiling to clarify the biological drivers of GC-EN and identify therapeutic targets. Prospective studies are required to evaluate systemic therapies, including chemotherapy and immunotherapy, in this population. The development of GC-EN-specific prognostic models and international collaboration are critical for establishing tailored treatment strategies for this rare but aggressive disease.

## 5. Conclusions

GC-EN remains uncommon but carries a clear survival penalty. The institutional proportion of GC-EN decreased over time, whereas the SEER proportion increased slightly with minor fluctuations. In recent years, survival outcomes for patients with NEC at our center appeared to improve, but this temporal improvement should be interpreted cautiously because patient-level treatment and screening data were not collected and follow-up maturity differed across periods. NEC-dominant subgroup is the major driver of poor prognosis within GC-EN, whereas MiNEN and AC-dominant tumors showed survival curves similar to those of GC in this cohort. These findings support GC-EN-specific risk stratification according to the neuroendocrine carcinoma component and highlight the need for future studies incorporating systemic treatment records to guide tailored management.

## Figures and Tables

**Table 1:** Baseline information between gastric carcinoma with coexisting exocrine and neuroendocrine components (GC-EN) and gastric cancer (GC) after pTNM stage matching.

		GC-EN Group	GC Group	*p* Value
		*n* (%)	*n* (%)	
**Gender**				<0.01
	Male	251 (85%)	440 (75%)	
	Female	43 (15%)	148 (25%)	
**Age, years**				<0.01
	<40	7 (2.4%)	11 (1.9%)	
	40–65	183 (62%)	524 (89%)	
	>65	104 (35%)	53 (9.0%)	
**Tumor size (cm)**				0.03
	<4	108 (37%)	261 (44%)	
	>4	186 (63%)	327 (56%)	
**Tumor location**				<0.01
	Proximal	186 (63%)	195 (33%)	
	Distal	101 (34%)	309 (53%)	
	Overlapping lesions	7 (2.4%)	84 (14%)	
**Number of lymph node metastases**				0.8
		5.90 (9.37)	5.03 (7.97)	
**Number of lymph nodes retrieved**				<0.01
		33.02 (15.74)	29.52 (15.09)	
**Signet ring**				<0.01
	No	275 (94%)	436 (74%)	
	Yes	19 (6.5%)	152 (26%)	
**HER2 score**				<0.01
	0 (−)	229 (78%)	335 (57%)	
	1 (+)	33 (11%)	159 (27%)	
	2 (++)	25 (8.5%)	67 (11%)	
	3 (+++)	7 (2.4%)	27 (4.6%)	
**P stage**				>0.9
	I	33 (11%)	66 (11%)	
	II	75 (26%)	150 (26%)	
	III	186 (63%)	372 (63%)	
**Vascular invasion**			<0.01	
	No	130 (44%)	362 (62%)	
	Yes	164 (56%)	226 (38%)	
**Perineural invasion**				0.6
	No	162 (55%)	314 (53%)	
	Yes	132 (45%)	274 (47%)	
**Complication**				0.6
	No	264 (90%)	534 (91%)	
	Yes	30 (10%)	54 (9.2%)	

Note: GC tumor–node–metastasis (TNM) stage according to the 8th edition of the American Joint Committee on Cancer (AJCC) staging system. HER2 expression was semi-quantitatively scored by immunohistochemistry as negative (−), weak positive (+), moderate positive (++), or strong positive (+++).

**Table 2:** Overall Survival of NEC (NCC vs. SEER).

	1-Year Survival	2-Year Survival	3-Year Survival	5-Year Survival
NCC	88.6% (83.3–93.1%)	68.3% (61.3–75.7%)	44.9% (37.9–53.8%)	42.7% (33.3–49.2%)
SEER	65.5% (63.4–67.6%)	53.2% (51–55.5%)	47.8% (45.5–50.1%)	41% (38.8–43.4%)

NCC, National Cancer Center; SEER, surveillance, epidemiology, and end results.

**Table 3:** Univariate and multivariate Cox proportional hazards regression analyses for overall survival in patients with gastric carcinoma with coexisting exocrine and neuroendocrine components (GC-EN) and gastric cancer (GC) at China National Cancer Center.

Factors		Univariate Analysis			Multivariate Analysis *		
		Hazard Ratio	95% CI	*p* Value	Hazard Ratio	95% CI	*p* Value
**Group**							
	GC	1.00			1.00		
	GC-EN	1.66	1.34–2.05	<0.01	1.49	1.15–1.92	<0.01
**Gender**							
	Male	1.00					
	Female	1.10	0.86–1.41	0.44			
**Age, years**							
	<40	1.00					
	40–65	0.35	0.19–0.62	<0.01	0.40	0.22–0.72	<0.01
	>65	0.55	0.30–1.01	0.06			
**Tumor size(cm)**							
	<4.0	1.00			1.00		
	≥4.0	1.46	1.18–1.82	<0.01	1.06	0.85–1.34	0.59
**Tumor location**							
	Proximal	1.00					
	Distal	0.78	0.62–0.97	0.03			
	Overlapping lesions	1.01	0.71–1.63	0.42			
**Number of lymph nodes retrieved, per node**		0.99	0.98–1.00	<0.01	0.98	0.97–0.99	<0.01
**Metastatic lymph nodes, per node**		1.03	1.02–1.04	<0.01	1.03	1.02–1.05	<0.01
**Signet ring**							
	No	1.00					
	Yes	1.05	0.80–1.36	0.72			
**HER2 score**							
	0 (−)	1.000					
	1 (+)	0.80	0.61–1.04	0.09			
	2 (++)	0.71	0.48–1.03	0.07			
	3 (+++)	1.15	0.69–1.91	0.59			
**pTNM stage**							
	I	1			1.00		
	II	4.20	2.02–8.47	<0.01	4.54	2.17–9.50	<0.01
	III	8.07	3.99–16.3	<0.01	7.35	3.58–15.1	<0.01
**Vascular invasion**							
	No	1.000					
	Yes	1.28	1.04–1.58	0.02	0.97	0.75–1.24	0.80
**Perineural invasion**							
	No	1.000					
	Yes	0.92	0.74–1.13	0.42			

* Variables with a *p*-value < 0.05 in the univariate analysis were ultimately included in the multivariate analysis. The numbers of retrieved lymph nodes and metastatic lymph nodes were modeled as continuous variables. HRs represent the change in hazard per one additional lymph node. TNM, tumor–node–metastasis. HER2 expression was semi-quantitatively scored by immunohistochemistry as negative (−), weak positive (+), moderate positive (++), or strong positive (+++).

## Data Availability

De-identified data may be obtained from the corresponding author upon reasonable request and with appropriate justification, subject to privacy restrictions.

## Supplementary Materials

The following supporting information can be downloaded at: https://www.mdpi.com/article/10.3390/curroncol33090558/s1, Table S1: Yearly share of NEC among GC in NCC; Yearly share of NEC among GC in SEER; Table S2: Survival of NEC by period in NCC; Survival of NEC by period in SEER; Table S3: Comparison of NEC Survival Rates Between Our Center and Other Foreign Medical Institutions.

## Abbreviations

The following abbreviations are used in this manuscript:

GC	Gastric cancer
GC-EN	Gastric carcinoma with coexisting exocrine and neuroendocrine components
GNEC	Gastric neuroendocrine carcinoma
NEC	Neuroendocrine carcinoma
MANEC	Mixed adenoneuroendocrine carcinoma
MiNEN	Mixed neuroendocrine–non-neuroendocrine neoplasm
AC-dominant	Adenocarcinoma-dominant
NCC	National Cancer Center
SEER	Surveillance, Epidemiology, and End Results
OS	Overall survival
KM	Kaplan–Meier
HR	Hazard ratio
CI	Confidence interval
Cox model	Cox proportional hazards regression model
TNM	Tumor–node–metastasis
AJCC	American Joint Committee on Cancer
ICD-O-3	International Classification of Diseases for Oncology, Third Edition
NOS	Not otherwise specified
HER2	Human epidermal growth factor receptor 2
PNI	Perineural invasion
LVI	Lymphovascular invasion
VI	Vascular invasion
LN	Lymph node
