# Supplementary material for: Gastric Carcinoma with Exocrine and Neuroendocrine Components: A 16-Year Cohort Analysis and International Comparison
Source: Curr Oncol. 2026 Sep 15;33(9):558. doi: 10.3390/curroncol33090558 (PMC13605138; doi:10.3390/curroncol33090558)
Supplement: Supplementary file 1 [file curroncol-33-00558-s001.zip › curroncol-4530622-supplementary.pdf]

Supplemental Table S1

Yearly share of NEC among GC in NCC

year（ncc）	GC(n)	NEC(n)	proportion
2008-2009	349	14	4.01%
2010-2011	479	21	4.38%
2012-2013	569	23	4.04%
2014-2015	1200	27	2.25%
2016-2017	1420	22	1.55%
2018-2019	1930	25	1.30%
2020-2021	1638	19	1.6%

Yearly share of NEC among GC in SEER

year（SEER）	GC(n)	NEC(n)	proportion
2008-2009	9816	164	1.67%
2010-2011	9993	217	2.17%
2012-2013	10275	279	2.72%
2014-2015	10400	234	2.25%
2016-2017	10364	255	2.46%
2018-2019	10350	274	2.65%
2020-2021	10055	245	2.44%

Supplemental Table S2

Survival of NEC by period in NCC			
	1-year overall survival	2-year overall survival	3-year overall survival
-2012	85.1%	59.6%	34%
2013-2016	85.7%	61.2%	32.7%
2017-2020	91.9%	73%	58.9%
2021-2024	94.1%	87.5%	76.2%

Survival of NEC by period in SEER			
	1-year overall survival	2-year overall survival	3-year overall survival
-2012	71.3%	58.7%	53.4%
2013-2016	68.9%	58.2%	51.8%
2017-2020	65%	52.9%	48.6%
2021-2024	66.9%		

Supplemental Table S3

Comparison of NEC Survival Rates Between Our Center and Other Foreign Medical Institutions

	1-year overall survival	2-year overall survival	3-year overall survival	5-year overall survival
NCC	88.6%(83.3%–93.1%)	68.3% (61.3%–75.7%)	44.9% (37.9%–53.8%)	42.7% (33.3%–49.2%)
rome、parma	37.70%	19.70%	16.50%	9.70%
USA，Seer	65.80%	51.30%	45.60%	39.10%
Berlin in Germany	74.60%	61.30%	44.40%	33.70%
